# Azithromycin Synergistically Enhances Anti-Proliferative Activity of Vincristine in Cervical and Gastric Cancer Cells

**DOI:** 10.3390/cancers4041318

**Published:** 2012-12-04

**Authors:** Xuezhang Zhou, Yuyan Zhang, Yong Li, Xiujing Hao, Xiaoming Liu, Yujiong Wang

**Affiliations:** 1 Key Laboratory of the Ministry of Education for the Conservation and Utilization of Special Biological Resources of Western China, Yinchuan 750021, Ningxia, China; E-Mails: zhouxuezhang@yahoo.com.cn (X.Z.); yujiongw@gmail.com (Y.Z.); liyong7732@126.com (Y.L.); lxiaom66@hotmail.com (X.H.); 2 College of Life Science, Ningxia University, Yinchuan 750021, Ningxia, China

**Keywords:** azithromycin, macrolides, vincristine, cytotoxicity, apoptosis, cancer cells

## Abstract

In this study, the anti-proliferative and anticancer activity of azithromycin (AZM) was examined. In the presence of AZM, cell growth was inhibited more effectively in Hela and SGC-7901 cancer cells, relative to transformed BHK-21 cells. The respective 50% inhibition of cell growth (IC_50_) values for Hela, SGC-7901 and BHK-21 were 15.66, 26.05 and 91.00 µg/mL at 72 h post incubation, indicative of a selective cytotoxicity against cancer cells. Cell apoptosis analysis using Hoechst nuclear staining and annexin V-FITC binding assay further demonstrated that AZM was capable of inducing apoptosis in both cancer cells and transformed cells. The apoptosis induced by AZM was partly through a caspase-dependent mechanism with an up-regulation of apoptotic protein cleavage PARP and caspase-3 products, as well as a down-regulation of anti-apoptotic proteins, Mcl-1, bcl-2 and bcl-X1. More importantly, a combination of AZM and a low dose of the common anti-cancer chemotherapeutic agent vincristine (VCR), produced a selectively synergistic effect on apoptosis of Hela and SGC-7901 cells, but not BHK-21 cells. In the presence of 12.50 μg/mL of VCR, the respective IC_50_ values of Hela, SGC-7901 and BHK-21 cells to AZM were reduced to 9.47 µg/mL, 8.43 µg/mL and 40.15 µg/mL at 72 h after the incubation, suggesting that the cytotoxicity of AZM had a selective anti-cancer effect on cancer over transformed cells *in vitro*. These results imply that AZM may be a potential anticancer agent for use in chemotherapy regimens, and it may minimize side effects via reduction of dosage and enhancing the effectiveness common chemotherapeutic drugs.

## 1. Introduction

Antibiotics are generally thought to be safe agents with trivial or no side effects, since they typically use unique molecular targets against microorganisms. Furthermore, aside from their anti-microbial activity properties, several classes of antibiotics display an ability to induce cell apoptosis, which is believed to be an important property of any potential anti-cancer drug [1,2,3,4,5].

Azithromycin (AZM), a 15-ring member macrolide antibiotic, differs from other macrolides known to inhibit cytochrome P450 (CYP) 3A4 [6]. This unique metabolic feature leads AZM to possess the favorable property of rapidly accumulating and slowly releasing into cells and tissues, resulting in a higher local concentration of drug, and a longer elimination half-life [7]. Like other macrolides, apart from its antimicrobial properties, AZM also exhibits anti-inflammation, immunomodulation, and anti-proliferation effects, as well as an autophagic effect by leading to apoptotic cell death [8,9]. AZM has shown an ability to induce neutrophil apoptosis, as well as inhibit proliferation of peripheral blood mononuclear cells [10]; these have been suggested to contribute the anti-inflammatory activity induced by AZM [11,12,13]. Additionally, AZM has shown an ability to suppress the proliferation of tracheal smooth muscle cells (SMCs) through a mechanism of induction of apoptotic cell death [8], and reverse resistance of P-glycoprotein-dependent anticancer drugs *in vitro* [14]. However, the effect of AZM on cancer cell apoptosis and its interactions with commonly used anti-cancer agents have not been investigated yet.

Vincristine (VCR), the most toxic of the vinca alkaloids, is a mitotic inhibitor with a cytotoxicity due to interference with microtubule formation and mitotic spindle dynamics, that eventually disrupts the cells via a cell apoptosis mechanism [15]. VCR currently remains an invaluable agent used in many cancer chemotherapy regimens. However, the clinically important adverse effect of VCR-induced neutrotoxicity have limited its potential effectiveness in cancer therapy, which restricts the physician to use of a suboptimal dose in many patients where the effectiveness of VCR has been proved [16,17]. In order to overcome the toxicity and resistance of chemotherapeutic agents, and to enhance their efficacy, combinational strategies with multiple agents including antibiotics are being studied. For example, rapamycin and rapamycin analogs have been tested in combination with other standard chemotherapeutic agents, and they have shown to be a promising option for the treatment of cancers [18,19,20].

The aim of this study was to determine whether AZM was able to induce cancer cell apoptosis and enhance the efficacy of the commonly used chemotherapeutic agent VCR in cancer cell lines and a transformed cell line *in vitro*.

## 2. Results and Discussion

### 2.1. Effect of AZM and VCR on Viability of Various Cells

**Figure 1:**
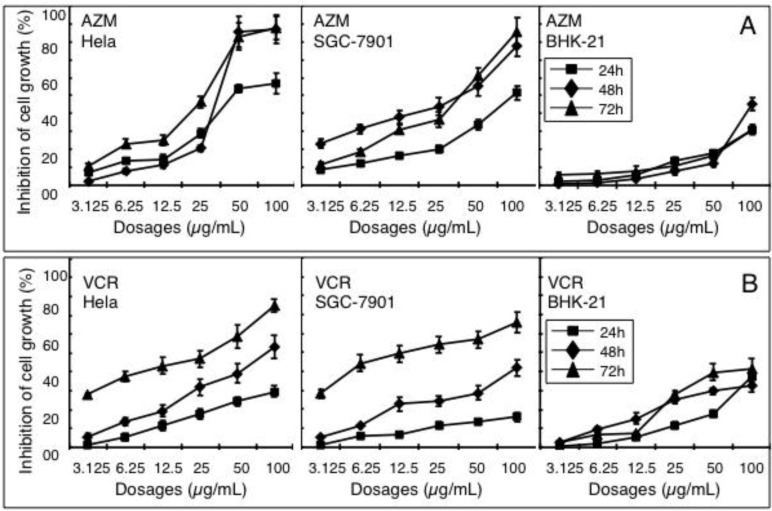
Dose-dependent inhibition of cell proliferation induced by azithromycin (AZM) and vincristine (VCR). Hela, SGC-7901 and BHK-21 cells were incubated with different doses of AZM (**A**) and VCR (**B**) for indicated time periods. The inhibitory effects of drugs on cell growth were determined by accessing cell viability using an MTT assay. Results represent the mean (±SD) of nine samples (N = 9) from three independent experiments for each condition.

An MTT viability assay was performed to determine the anti-proliferative activity of AZM and VCR in cancer cells by evaluating the inhibition of cell growth. Hela cells (a cervix adenocarcinoma cell line), SGC-7901 cells (a gastric cancer cell line) and BHK-21 (a transformed hamster fibroblast cell line) were treated with various concentrations of AZM or VCR alone, of doses ranging from 3.13 to 100 µg/mL. A significant inhibition of cell growth was observed in all tested cell types treated with AZM or VCR alone (*p* < 0.05, Figure 1), and these inhibitions were time and dose-dependent.

Notably, both AZM and VCR (particularly the AZM) exhibited an ability to selectively induce cancer cell death, in which they were more effective at inhibiting the proliferations of Hela and SGC-7901 cells than that of BHK-21 cells, a transformed fibroblast cell line. Additionally, AZM showed more inhibitory effects on these two cancer cell lines, in comparison with the VCR, one of the most commonly used chemotherapy drugs, when higher doses of agents (≥50 µg/mL) were applied, particularly in the Hela cells (Figure 1). This result indicated AZM has a much higher activity towards cancer cells. The concentration of compound required for a 50% inhibition of cell growth (IC_50_) was obtained by extrapolation from an inhibition curve. In the presence of AZM alone, the respective IC_50_ values of Hela cells were 44.78, 25.09 and 15.66 µg/mL at 24, 48 and 72 h after the incubation; the IC_50_s of SGC-7901 cells were 41.90, 21.74 and 26.05 µg/mL at 24, 48 and 72 h following the treatment, respectively; the IC_50_s of BHK-21 cells were 95.70, 95.02 and 91.00 µg/mL at 24, 48 and 72 h post incubation (Figure 1A). In the presence of VCR alone, the IC_50_ values of Hela cells were 85.31, 49.17 and 18.39 µg/mL at 24, 48 and 72 h after the incubation, respectively; the IC_50_ values of SGC-7901 cells were 111.63, 61.26 and 17.99 µg/mL at 24, 48 and 72 h after the treatment, respectively; and the respective IC_50_s of BHK-21 cells were 94.18, 71.73 and 65.89 µg/mL at 24, 48 and 72 h post drug exposure (Figure 1B). Of note, a lower inhibition at 72 h or 48 h than 24 h or 48 h was observed when some types of cells were treated with a higher dose of agents, probably due in part to the loss of some detached cells in this type of cell at the concentration applied for longer time period, during the processing of MTT assay.

### 2.2. Synergistic Effect of a Combination of AZM and VCR on the Proliferation of Cells

The growth of Hela, SGC-7901 and BHK-21 cells treated with a combination of VCR and AZM was determined by an MTT assay and coefficient of drug interaction (CDI) analysis. The inhibition efficiency was used to test the effectiveness of a combination of AZM and VCR in each of the cell lines. The dosage used for the cell lines was 12.5 μg/mL of VCR with increasing doses of AZM (covering a range from 3.13 to 100 µg/mL, Table 1). A beneficial inhibitory effect of a simultaneous use of both agents was observed in all the three tested cell lines. In most of the tested groups, cells exposed to a combination of VCR (12.5 µg/mL) and various doses of AZM, evidenced a synergistic effect which was determined by the CDI analysis (CDI < 1.0), where the proliferation of cells exposed to a combination of drugs was significantly inhibited than that by each drug alone. This was particularly seen in the cancer cells (SGC-7901 and Hela) exposed to a combination of VCR with a lower dose of AZM (*p* < 0.05, Table 1).

**Table 1:** Synergistic inhibition of VCR and AZM on cancer cell lines (%) (Mean ± SD) ^a^.

Cell lines	VCR/AZM (µg/mL)	24 h		48 h		72 h	
		Inhibition (%)	CDI	Inhibition (%)	CDI	Inhibition (%)	CDI
Hela	12.5/100	55.690 ± 0.707 ^**^	0.87	70.197 ± 0.404 ^*^	0.66	84.444 ± 0.652 ^**^	1.22
	12.5/50	44.062 ± 0.525 ^**^	0.94	66.565 ± 0.484 ^*^	0.65	75.021 ± 0.572 ^**^	1.55
	12.5/25	41.527 ± 0.777 ^**^	0.98	61.630 ± 0.623 ^*^	0.76	61.425 ± 0.610 ^**^	1.26
	12.5/12.5	35.326 ± 0.841 ^*^	0.98	52.980 ± 0.730 ^*^	0.88	56.577 ± 0.506 ^*^	0.97
	12.5/6.25	32.461 ± 0.510 ^**^	0.88	47.519 ± 0.423 ^**^	0.91	54.743 ± 0.525 ^**^	0.89
	12.5/3.125	30.063 ± 0.565 ^**^	0.82	39.978 ± 0.620 ^*^	0.83	49.810 ± 0.431	0.94
	12.5/0	11.753 ± 0.819	-	19.322 ± 0.943	-	43.143 ± 0.476	-
SGC-7901	12.5/100	60.884 ± 1.854 ^**^	0.86	71.556 ± 0.353 ^**^	0.95	75.716 ± 1.330 ^**^	1.78
	12.5/50	56.059 ± 1.798 ^**^	0.69	69.255 ± 0.718 ^**^	0.89	71.356 ± 0.574 ^**^	1.45
	12.5/25	52.855 ± 1.718 ^**^	0.63	63.739 ± 0.528 ^**^	0.84	69.574 ± 1.082 ^**^	0.93
	12.5/12.5	51.639 ± 3.291 ^**^	0.62	61.856 ± 0.736 ^**^	0.69	66.805 ± 0.415 ^*^	0.96
	12.5/6.25	49.724 ± 2.047 ^**^	0.63	57.830 ± 0.880 ^**^	0.64	60.630 ± 1.010 ^*^	0.90
	12.5/3.125	44.383 ± 1.842 ^**^	0.65	56.314 ± 0.753 ^**^	0.65	54.965 ± 0.921	0.92
	12.5/0	6.432 ± 0.498	-	22.739 ± 0.697	-	49.418 ± 0.547	-
BHK-21	12.5/100	50.707 ± 0.652 ^**^	0.75	74.284 ± 0.546 ^**^	0.55	65.247 ± 0.649 ^**^	0.54
	12.5/50	19.801 ± 0.602 ^**^	1.03	53.194 ± 0.551 ^**^	0.63	39.077 ± 0.404 ^**^	0.79
	12.5/25	13.271 ± 0.549 ^*^	1.06	25.523 ± 0.702	1.00	29.740 ± 0.579 ^**^	0.85
	12.5/12.5	8.426 ± 0.757	1.02	16.823 ± 0.663	1.02	24.174 ± 0.842 ^**^	0.89
	12.5/6.25	5.146 ± 0.538	1.03	12.225 ± 0.547	1.05	21.468 ± 0.345 ^*^	1.01
	12.5/3.125	2.708 ± 0.644	1.05	2.561 ± 0.799	1.16	8.383 ± 0.418	1.05
	12.5/0	5.360 ± 0.620	-	15.154 ± 0.823	-	7.623 ± 0.650	-

^a^: Compared with 12.5 µg/mL of VCR, ^*^: *p* < 0.05; ^**^: *p* < 0.01.

In the presence of 12.5 μg/mL of VCR, the IC_50_ values of Hela cells to AZM were 27.57, 13.26 and 9.47 µg/mL at 24, 48 and 72 h after the treatment, respectively; the IC_50_ values of SGC-7901 cells to AZM were 16.02, 9.63 and 8.43 µg/mL at 24, 48 and 72 h post the incubation, respectively; the IC_50_ values of BHK-21 cells to AZM were 76.53, 48.90 and 40.15 µg/mL at 24, 48 and 72 h after the incubation, respectively. This data suggests that a combination of AZM and VCR displays synergy and increases the inhibition of cancer cell proliferation.

### 2.3. Induction of Cell Apoptosis by AZM and VCR

**Figure 2:**
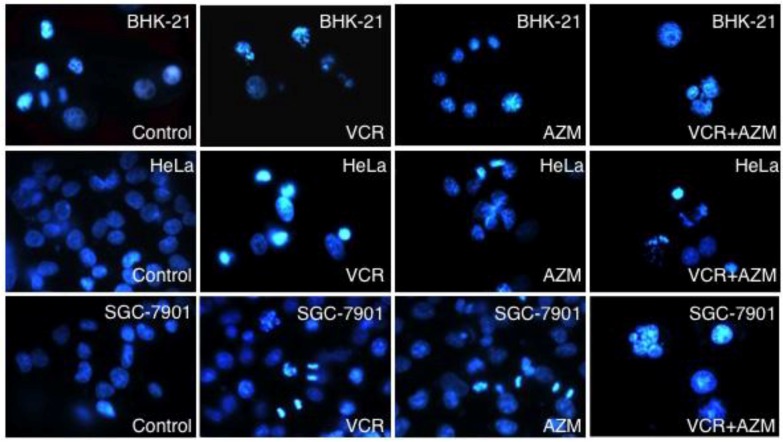
Nuclear morphology monitored by Hoechst dye staining. Cell of indicated cell types were grown in a medium containing each 12.5 µg/mL of AZM or VCR alone, or a combination of the two drugs for 48 h, and then stained with dye Hoechst 33258. Chromatin structure was observed under a fluorescence microscope.

**Figure 3:**
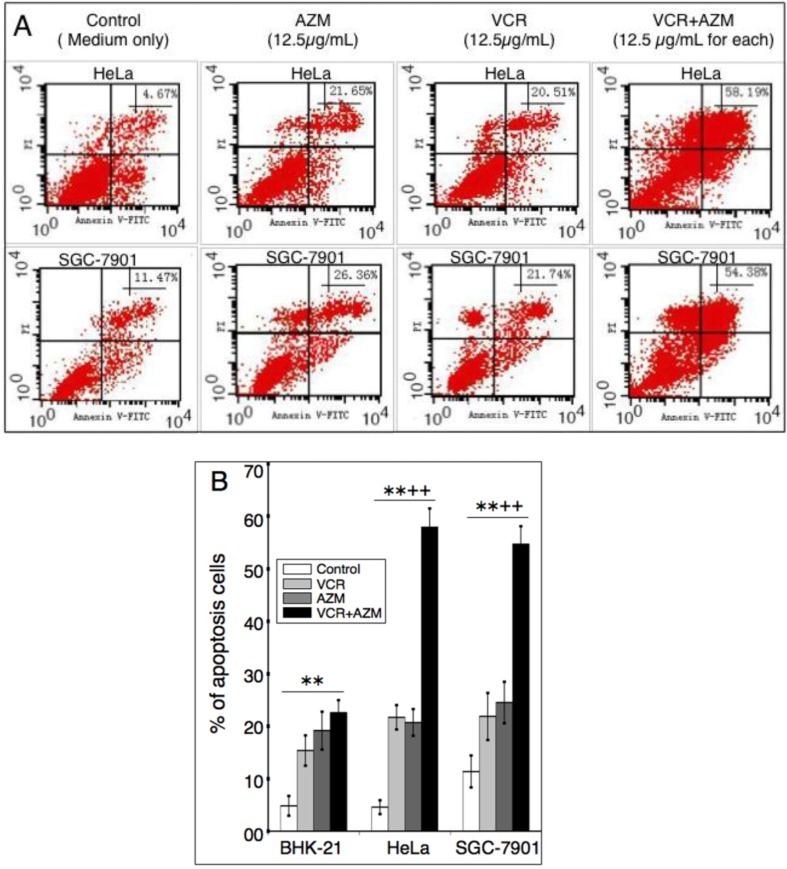
Apoptosis determined by annexin V binding assay and flow cytometry analysis. Indicated cells were treated with 12.5 µg/mL of each AZM or VCR alone, or a combination of the two agents for 48 h, and then stained with annexin V, followed by cytometry analysis. (**A**) A representative profile of Annexin-V-FITC/PI staining of Hela cells (top panel) and SGC-7901 cells treated with indicated agents for 48 h. The bottom left quadrant shows the viable cells, neither binding annexin V nor showing PI uptake; the bottom right quadrant represents the early apototic cells, binding annexin V but still retaining their cytoplasmic integrity and excluding PI; the top left quadrant shows nonviable necrotic cells/nuclear fragments with no annexin V binding but PI uptake; the top right quadrant represents nonviable, late apoptotic/necrotic cells, positive for annexin V and PI staining, the percentage of cells falling within this corner was indicated. (**B**) Statistical analysis of results of nine samples. Data represent the mean (±SD) from three independent experiments for each condition (N = 9). Compared with medium control, **: *p* < 0.01; compared with each of AZM or VCR alone, ^++^: *p* < 0.01.

We next sought to determine if apoptosis was responsible for the inhibition of cell proliferation induced by AZM and VCR. Cells were treated with 12.5 µg/mL of each of AZM or VCR alone, or in combination for 24 or 48 h, and apoptosis was first assessed by Hoechst staining and observation of nuclear morphology, annexin V-FITC binding and cytometry (Figure 2 and Figure 3).

Consistent with the characteristics of cell apoptosis [21], both cancer cells (Hela and SGC-7901) and transformed cells (BHK-21) exposed to a low concentration (12.5 µg/mL) of AZM, VCR or a combination of these two drugs exhibited morphology of nuclear chromatin condensation and fragmentation with significant levels of bright, punctuate nuclei, following the Hoechst staining (Figure 2 and data not shown). The apoptosis of cells was further ascertained by annexin V-FITC/propidium iodide (PI) binding and cytometry analysis. This method is able to distinguish early apoptotic cells (annexin V-FITC positive only) from late apoptotic or necrotic cells (annexin V-FITC and PI double positive). Each agent alone was capable of significantly inducing cell apoptosis/necrosis in a time-dependent manner, in comparison with those cells exposed to mock control media (*p* < 0.01) (Figure 3 and data not shown). Importantly, a combination of AZM and VCR induced significant apoptosis/necrosis in cancer cells relative to each agent alone (*p* < 0.01, Figure 3B). Moreover, such a significantly synergistic effect in induction of apoptosis was not observed in the transformed BHK-21 cell line (Figure 3B). Annexin V-FITC binding and cytometry analysis revealed that apoptotic/necrotic populations of Hela cells incubated with control, AZM, VCR and a AZM/VCR combination for 48 h, were (4.59 ± 1.32)%, (20.71 ± 2.55)%, (21.72 ± 2.32)% and (57.92 ± 3.55)%, respectively; the respective apoptotic populations of SGC-7901 cells treated with control, AZM, VCR and a combination AZM/VCR for 48 h, were (11.38 ± 3.05)%, (24.52 ± 3.97)%, (21.88 ± 49)% and (54.67 ± 3.44)%. These results implied that AZM was capable of synergistically interacting with VCR and selectively inducing cancer cell apoptosis/necrosis.

### 2.4. Effect on Caspase-3 Activity

**Figure 4:**
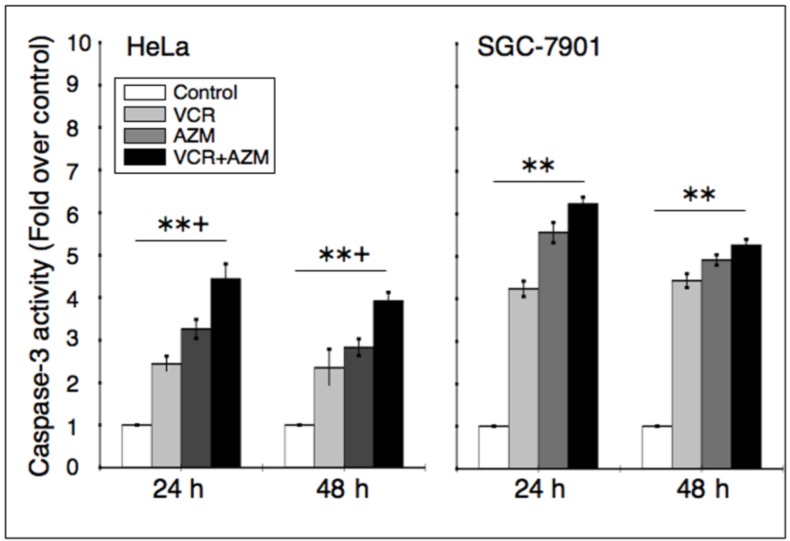
Effect of AZM or VCR on caspase-3 activity in cancer cells. Hela and SGC-7901 cells were treated with 12.5 µg/mL of each AZM or VCR alone, or a combination of the two agents for 24 and 48 h, the cell lysates were then employed for determining caspase-3 activity. Values represents fold of activity over the control lysates. The data express the mean (±SD) of nine samples from three independent experiments for each condition (N = 9). Compared with medium control, **: *p* < 0.01; compared with each of AZM or VCR alone, ^+^: *p* < 0.05.

To examine whether AZM-induced apoptosis involved an activation of caspase, cells were incubated with 12.5 µg/mL of each of AZM or VCR alone, or in combination for 24 or 48 h, and caspase-3 activity was evaluated using a chromogenic caspase-3 substrate. Caspase-3 activity was significantly enhanced in the cancer cells exposed to AZM or VCR alone and a combination of AZM/VCR by 2.5–4.5 fold in Hela cells, and 4.5–6.5 fold in SGC-7901 cells, in comparison with the medium controls (Figure 4). Additionally, the activity of caspase-3 in Hela cells was significantly enhanced, as compared with each of agent alone (right panel in Figure 4). This result indicates that activation of the caspase cascade may at least in part contribute to the ability of AZM and VCR to induce cancer cell apoptosis.

### 2.5. Effect on the Expression of Apoptotic Proteins

**Figure 5:**
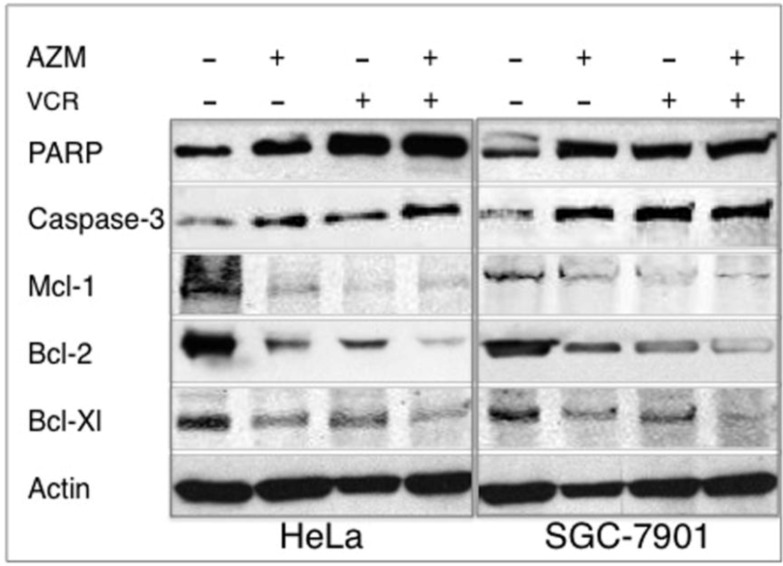
Western blotting analysis of expressions of apoptotic protein. Hela and SGC-7901 cells incubated in a medium containing each 12.5 µg/mL of AZM or VCR alone, or a combination of the two drugs for 48 h, the cell lysates were then used for analyzing the alteration of expressions of apoptotic and anti-poptotic proteins by immunoblotting analysis against indicated antibodies. The experiments were repeated three times and similar results were found.

Poly(ADP-ribose)polymerase (PARP) is a classical caspase substrate, and the proteolytic cleavage of PARP and activation of caspase-3 are a hallmark of apoptosis. In order to understand the mechanism of AZM induced cancer cell apoptosis, the expression of apoptotic proteins (PARP and caspase-3), and anti-apoptotic proteins (Mcl-1, bcl-2 and bcl-X1) were ascertained by immunoblotting assay (Figure 5). Hela and SGC-7901 cells treated with 12.5 µg/mL of AZM, VCR or in combination for 48 h showed increased expressions of apoptotic protein, cleavage PARP and caspase-3 products, as well as decreased expressions of anti-apoptotic proteins, Mcl-1, bcl-2 and bcl-X1 (Figure 5), indicative of the ability of each drugs alone or in combination inducing cancer cell apoptosis.

### 2.6. Discussion

Most currently used anti-cancer agents are toxic, and they may eventually lead to cancer-resistance to cancer treatment due to repeated application, which is a major cause of cancer treatment failure. Therefore, searching for agents capable of selectively targeting cancer cells *versus* normal cells is a primary goal in anti-cancer drug discovery. In the present study, we explored the cytotoxic effects of AZM and induction of apoptosis in cancer cells by itself or in combination with a commonly used anti-cancer chemotherapeutic agent, VCR. Our results showed that AZM had a selective cytotoxic property to cervical cancer Hela cells and gastric cancer SGC-7901 cells. Moreover, in combination with VCR, AZM exhibited the ability to mediate a synergistic effect on apoptosis of the tested cancer cells, but not the transformed BHK-21 cell line. The apoptosis induced by AZM was in part through a caspase-dependent mechanism, with an up-regulation of apoptotic protein PARP and caspase-3, and a down-regultialation of anti-apoptotic proteins, Mcl-1, bcl-2 and bcl-X1. These data suggest that AZM has a preferably anti-cancer activity on cancer cells over normal cells *in vitro*.

In addition to their known antimicrobial activities, a variety of antibiotics also are revealed to have alternative activities including anti-cancer properties. Of them, the mammalian target of rapamycin, a lipophilic macrolide antibiotic, has been most extensively investigated, and the therapeutic intervention targeting mTOR signaling has led the development of a number of mTOR inhibitors tested in clinical trails [22]. A group of β-lactam antibiotics, *N*-thiolated β-lactams, and some members of the macrolide antibiotic family, have also been demonstrated to have a selective anti-proliferative activity against human cancer cells but not normal cells, through a caspase-dependent cell apoptosis pathway [2,17,23,24,25,26,27]. Considering that most currently used anti-cancer agents were first discovered as antibiotics, and clinical anticancer therapy is often accompanied with an antibiotic treatment [28], a regimen using a combination of antibiotics with anti-cancer agents may enhance effectiveness and reduce doses of individual agents. This can reduce the side effects in the treatment of cancer. A number of studies have demonstrated that combinations of mTOR inhibitor, such as rapamycin, with other chemotherapeutic agents exhibited synergistic or additive effects on the induction of apoptosis *in vitro*, as well as potential for overcoming chemoresistance [18,29,30]. For example, rapamycin derivative, everolimus was able to increase the susceptibility of human lung cancer cells to cisplatin and enhance the cisplatin-induced apoptosis in these cells [30].

The capacity of inducing apoptosis and selectively targeting cancer cells has long been a focus for development of anti-cancer drugs. The potential apoptotic effect of AZM has been examined in neutrophils, peripheral blood mononuclear cells and tracheal smooth muscle cells [8,10,11]. In agreement with these findings, AZM displayed the ability to induce apoptosis of Hela, SGC-7901 and BHK-21 cells at a comparable degree *in vitro*, as determined by an annexin V-FITC binding assay (Figure 3B), although it showed a preferential anti-proliferative activity to cancer over transformed cells (Figure 1A). This implies that cyto-reductive mechanisms other than the induction of apoptosis may also be involved in the anti-cancer properties of AZM. A combination of AZM and VCR significantly increased the anti-proliferative activity and apoptotic cell population relative to the sum of the treatments of single agents (Figure 3) and exhibited CDI < 1.0 (Table 1), indicative of a synergistic effect. Importantly, significant synergy was only observed in cancer cells, but not found in the BHK-21 transformed cell line, in which AZM only demonstrated a minimal synergistic effect. This data suggest that the antibiotic AZM and the chemotherapeutic agent VCR may have a selectively synergistic relationship against cancer cells, despite the fact that the precise mechanism of interaction between them remains elusive.

The anticancer activity of macrolides has been previously determined in the 14-membered ring macrolide antibiotics, clarithromycin, roxithromycin and erythromycin. Clarithromycin had a therapeutically beneficial anticancer activity in mammary adenocarcinoma, and it enables prolonged median survival times in patients with nonresectable non-small cell lung cancer [31,32]; erythromycin was able to enhance the tumoricidal activity of macrophages and natural killer cells [33]; both clarithromycin and roxithromycin exhibit anticancer effects of reducing the tumor vascularity and increasing apoptosis of the cancer cells in the B16BL6 mouse melanoma and C57BL mouse system [26]. While studying the same model, AZM, and a 16-membered ring macrolide, josamycin, did not display any inhibitory effect on vascular angiogenesis and inhibition of tumor growth or pulmonary metastasis of B16BL6 cells *in vivo* [26]. This was different from the findings in our study, in which AZM exhibited anticancer effects in Hela and SGC-7901 cells *in vitro*, at least in part through a mechanism of inducing cell apoptosis. We reasoned that the dose-dependency, variety of mechanism and/or drug susceptibility of various tumors, might contribute to this discrepancy between the anticancer effects seen for different members of the macrolide class. Indeed, gastric cancer SGC-7901 cells showed more susceptibility to AZM relative to Hela cervical cancer cells when they were exposed to a low dose of this compound (<25 µg/mL, Figure 1A) in this report.

The major adverse side effect of chemotherapeutic agents, including VCR, is neurotoxicity, which can be potentiated through inhibition of P-glycoprotein or cytochrome P450 CYP 3A enzymes, the most important drug-metabolizing enzyme in the body [16,17]. The activity of inhibition of P-glycoprotein or CYP 3A may induce many drug interactions based on drug metabolism. For example, the macrolide erythromycin showed drug interactions with anti-cancer agents, including VCR [17]. Different from the 14-ring member macrolide antibiotics (erythromycin and clarithromycin) that are substrates of CYP 3A4 enzyme, AZM is not a substrate for CYP 3A4 and is unable to inhibit CYP 3A4 [6]. Such a metabolic property enabled AZM to reverse P-glycoprotein-dependent anticancer drug resistance *in vitro* [14]. The different metabolic responses between AZM and VCR might in part contribute the synergistic effect of these two agents seen in this study. This feature may provide a possibility to minimize VCR side effects by reducing the dosage.

## 3. Experimental Section

### 3.1. Reagents and Cell Lines

Chemicals used in this study were products of Sigma (St. Louis, MO, USA), unless otherwise indicated. Azithromycin (AZM, 99%) and vincristine (VCR, 95%) were also products from Sigma. 3-(4,5-dimethylthiazol-2-yl)-2,5-diphenyltetrazolium bromide (MTT) was obtained from Fluka (St. Louis, MO, USA). Hoechst-33258 Staining Kit was purchased from Beyotime Institute of Biotechnology (Beijing, China). Primary antibodies against Bcl-2, Mcl-1, cleavage PARP and HRP-Conjugated secondary antibodies were obtained from Beijing Biosynthesis Biotechnology (Beijing, China). Primary antibodies anti-Bcl-X_L_, β-actin and cleavage caspase-3 were purchased from Wuhan Boster Bio-Engineering Co. (Wuhan, China). The Caspase-3 activity Colorimetric Assay Kit was a product of Applygen Technologies Inc. (Beijing, China). Enhanced chemiluminescence (ECL) Western Blot Kit was purchased from Beijing ComWin Biotech (Beijing, China). Annexin V-fluorescein isothiocyanate (FITC) Apoptosis Detection Kit was a product of BD Biosciences (San Jose, CA, USA). Hela, SGC-7901 and BHK-21 cell lines were stocks of the Shanghai Cell Bank of the Chinese Academy of Sciences (Shanghai, China).

### 3.2. Cell Culture and Cell Proliferation Assay

Cells were cultured in RPMI-1640 media supplemented with 10% FBS and penicillin (100 IU/mL)/streptomycin (100 µg/mL) at 37 °C with humidified 5% CO_2_. The cytotoxic activity of agents (AZM and VCR, which were dissolved in dimethylsulfoxide (DMSO)) was determined by assessing the cell viability using an MTT colorimetric assay [34]. Briefly, Hela, SGC-7901 and BHK21 cells grown at a log phase were seeded in a 96 well at a density of (4–5) × 10^5^/well in 100 μL of culture media. The medium was replaced after 8 hours post-seeding with a new medium containing various concentrations of compounds and incubated for 24, 48 or 72 h. Control cultures were treated with DMSO. One hundred µL of 1 µg/mL MTT solution was then added to each well, and the cultures were incubated for additional 4 h. Followed by addition of 100 µL of 20% SDS in 50% dimethyl formamide and the formed crystals were dissolved by pipetting gently. Absorbance was measured at 550 nm using a microplate spectrophotometer. The cytotoxicity was ascertained by evaluating the median inhibitory concentration (IC_50_) value. The IC_50_ value represented the concentration of the compound at which cell growth was inhibited by 50%. The growth inhibition rate was calculated by the following formula:

Growth Inhibition (%) = (1 − average value of A_550_ nm from experimental groups/average A_550_ nm value of controls) × 100%

### 3.3. Analysis the Interaction of Compounds *in Vitro*

The coefficient of drug interaction (CDI) was used to analyze the synergistic inhibitory effects of drug combinations, which was calculated by following equation: CDI = AB/(A × B), where AB was the inhibitory ratio of the combination groups to control group; A or B was the ratio of the single agent group to control group. The CDI value <1, =1 and >1 represent a synergistic, additive and antagonistic effects, respectively. A less than 0.7 value of CDI indicates a significantly synergistic inhibitory effect [35,36].

### 3.4. Hoechst 33258 Staining

Apoptotic features in the cells were assayed by fluorescent staining with DNA-specific dye Hoechst 33258 (10 µM) for 30 min at 37 °C. The cells were washed three times with PBS, incubated in serum-free RPMI-1640 media, and the characteristic of chromatin condensation or nuclear fragmentation was observed under a fluorescence microscope (Leica, Wetzlar, Germany).

### 3.5. Annexin V-FITC/PI Staining

The Annexin V-FITC binding assay was employed for detection of cell apoptosis using an Annexin V-FITC Apoptosis Detection Kit detection kit per the manufacturer’s instructions. The cells grown at a log phase were seeded in a 6-well plate (1 × 10^6^/well) and treated at different conditions, then harvested. The cell pellets were resuspended in 400 µL of annexin V-FITC binding buffer (10 mM HEPES, 140 mM NaCl, 2.5 mM CaCl_2_, pH 7.4) containing a saturation concentration of annexin V-FITC and propidium iodide (PI), and incubated at room temperature (RT) for 15 min in the dark. The cells were then pelleted and analyzed in a BD FACS Calibur flow cytometer (Becton Dickinson, Franklin Lake, NJ, USA). Cells stained with annexin V-FITC and PI were considered as late apoptotic or necrotic cells, while cells stained with annexin V-FITC alone were considered early apoptotic cells. The fraction of cell population in different quadrants was analyzed using quadrant statistics.

### 3.6. Caspase-3 Activity Assay

Caspase-3 activity was analyzed using a Caspase-3 activity Colorimetric Assay Kit according to the manufacturer’s instructions. The cells were lysed in a buffer (10 mMTris (pH 7.5), 130 mM NaCl, 1% Triton X-100 (v/v), 10 mM NaPi, 10 mM NaPPi) for 60 min on ice. The lysate was centrifuged at 16,000 rpm at 4 °C for 15 min. The soluble fraction was collected and the protein concentration was determined. The caspase-3 activity assays were performed in 96-well microtitre plates by mixing the cell lysate and caspase assay buffer containing DEVD-pNA substrate and incubating at 37 °C for 2 h. Enzyme catalyzed release of pNA was accessed by monitoring the absorbance at 405 nm using a ELISA plate reader.

### 3.7. Western Blot Analysis

A whole cell extract was prepared by homogenizing the cells in a lysis buffer (50 mM Tris-HCl, pH 7.5, 5 mM EDTA, 150 mM NaCl, 0.5% NP-40) for 60 min on ice. The lysates were then centrifuged at 12,000 rpm for 10 min at 4 °C, and the supernatants were collected as whole-cell extracts. The soluble protein concentration was measured with Bio-Rad Protein Assay (Bio-Rad Laboratories, Richmond, CA, USA) using bovine serum albumin (BSA) as a standard. The cell extracts (100 µg) were separated by 10% sodium dodecyl sulfate (SDS)-polyacrylamide gel (SDS-PAGE) and transferred to a nitrocellulose membrane. The membrane was blocked in 4% nonfat dry milk in PBS containing 0.2% Tween-20 and probed using appropriate specific antibodies. The blots were developed using the enhanced chemiluminescence (ECL) reagent (Amersham Biosciences, Piscataway, NJ, USA).

### 3.8. Statistical Analysis

All data collected in this study was obtained from at least three independent experiments for each condition. SPSS v. 18.0 analysis software was used for the statistic analysis. Statistical evaluation of the data was performed by one-way ANOVA and t-test for comparison of differences between the two groups. A value *p* < 0.05 set to represent a statistical difference and a value *p* < 0.01 set to represent a significant difference. Data was presented as the mean ± standard deviations (SD).

## 4. Conclusions

Collectively, the present study reveals the anticancer activity of macrolide antibiotic azithromycin (AZM). Our results demonstrate that AZM possesses anti-proliferation and induction of apoptosis properties in cervical and gastric cancer cells. Furthermore, a combination of AZM and the chemotherapeutic drug vincristine (VCR) exhibits a likely selective synergistic effect on cancer cell apoptosis, partly through caspase activation. It is worthy to note that AZM is mainly used as an antibiotic, while VCR has been used as a classical cytotoxic drug for cancer therapy; the potential anticancer properties of AZM were only demonstrated in these two particular cell types *in vitro*. Nonetheless, pending further investigation AZM has shown a potential anticancer activity.
